# Coleopterans Associated with Plants that form Phytotelmata in Subtropical and Temperate Argentina, South America

**DOI:** 10.1673/031.011.14701

**Published:** 2011-11-07

**Authors:** Raúl E. Campos, Liliana A. Fernández

**Affiliations:** ^1^ Instituto, de Limnología “Dr. Raúl A. Ringuelet,” Universidad Nacional de La Plata - CONICET, CC 712 (1900) La Plata, Buenos Aires, Argentina; ^2^ Consejo Nacional de Investigaciones Científicas y Técnicas (CONICET)

**Keywords:** Aquatic beetle, *Aechmea distichantha*, biodiversity, *Eryngium* species, *Guadua* species

## Abstract

A list of the most common plants that form phytotelmata and their associated coleopterans (aquatic, semi-aquatic and terrestrial) from the northeastern subtropical and temperate area of Argentina, South America with biological and behavioral observations is presented in this study. Species of Poaceae (n = 3), Bromeliaceae (5), Apiaceae (6), Araceae (2), Urticaceae (1), Marantaceae (1), Arecaceae (1), Dipsacaceae (1) and Cyperaceae (1) were identified as phytotelmata. Aquatic species of Scirtidae (2), Dytiscidae (2), and Hydrophilidae (4), semi-aquatic Chelonariidae (2), and terrestrial species of Carabidae (3), Staphylinidae (5), Histeridae (1), Elateridae (1), Cantharidae (1), Cleridae (1), Tenebrionidae (1), Meloidae (1), Anthicidae (1), Chrysomelidae (3), Curculionidae (7) and Apionidae (1) were identified from six species of *Eryngium* L. (Apiales: Apiaceae), two species of *Guadua* Kunth (Poales: Poaceae), *Aechmea distichantha* Lemaire (Poales: Bromeliaceae), and from fallen leaves of *Euterpe edulis* Martius (Arecales: Arecaceae) from the temperate and subtropical area. The highest species richness was recorded in *Eryngium* phytotelmata. Fifteen species of beetles inhabit *Eryngium cabrerae* Pontiroli, 11 in *E. horridum* Malme, 7 in *E. stenophyllum* Urban, 4 in *E.* aff. *serra* Chamisso and Schlechtendal., 3 in *E. elegans* Chamisso and Schlechtendal, 2 in *E. eburneum* Decne and *E. pandanifolium* Chamisso and Schlechtendal. From bamboo, 6 species of coleopterans were collected from *Guadua trinii* (Nees) Nees ex Ruprecht and 4 from *G. chacoensis* (Rojas) Londoño and Peterson. Three species of aquatic coleopterans were recorded from *A. distichantha* and only one from *E. edulis.*

## Introduction

Phytotelmata are pools of water impounded by plants (Vargas 1928). The plants, for the most part, are living but the term phytotelmata also includes pools of water held ephemerally on concave surfaces of fallen leaves (Kitching 1971; Maguire 1971). A specific community develops in these micro environments, establishing food chains where not only aquatic species but also terrestrial visiting species are involved (Frank and Lounibos 1983). The phytotelmata are distributed in all continents except Antarctica, but diversity is greater in the tropics and subtropics. Approximately 1500 plant species where aquatic insects may find habitats suitable for their development (Fish 1983) are known. Among the most studied phytotelmata, the Bromeliaceae native to the New World, the Heliconiaceae from South and Central America and Indonesia, and the Araceae of cosmopolitan distribution are highlighted. Pitcher plants (Sarraceniaceae and Nepenthaceae) are special phytotelmata due to their insectivorous characteristic. However, some organisms are able to survive in their digestive fluid (Beaver 1983). A subset of phytotelmata is the so-called dendrotelmata, cavities in hollow trees and stumps of bamboo (Poaceae), some of which can hold water for a long time.

The phytotelmata support a large number of species, mainly insects belonging to the order Odonata, Plecoptera, Trichoptera, Hemiptera, Coleoptera and Diptera. The latter are found in all types of phytotelmata, as opposed to other orders (Fish 1983). Few identified species of beetles have been reported from phytotelmata, of which only members of the families Scirtidae, Dytiscidae, and Hydrophilidae are aquatic (Frank 1983; Kitching 1983). The other families are mainly terrestrial and at least 20 are known from the Neotropical and Nearctic regions (Mestre et al. 2001; Juncá and Da Silva Borges 2002; Frank et al. 2004), although only the species of Staphylinidae were studied (Frank and Barrera 2010).

These studies on aquatic beetles of Argentina were mostly taxonomic, but few were carried out on communities. We know of two studies involving communities of aquatic beetles of temperate and subtropical regions. One of them is about the structure and temporal change in the community of coleopterans inhabiting permanent, semi-permanent and temporary puddles (Von Ellenrieder and Fernández 2000). The other is an inventory of species from a National Park in the subtropical region, where new distributions and species from Argentina are reported (Fernández et al. 2008). A third study, not exclusively on aquatic coleopterans, refers to the community of organisms that live in ephemeral pools, referring to beetles as potential predators of a flood-water mosquito (Campos et al. 2004). There are no studies that consider species lists or communities of beetles that live in or are associated with phytotelmata in Argentina, except the study of Montero et al. (2010) which cites coleopterans to family level, associated with *Aechmea distichantha* Lemaire (Poales: Bromeliaceae).

During previous studies on mosquitoes that inhabit phytotelmata, associated beetles with each phytotelmata class were collected without performing an exhaustive search. A preliminary report of phytotelmata and their aquatic and amphibious beetles collected from subtropical and temperate Argentina is presented here. This study includes occasional terrestrial beetles that use the same plants as refuge.

## Materials and Methods

### Study area

**Figure 1.**
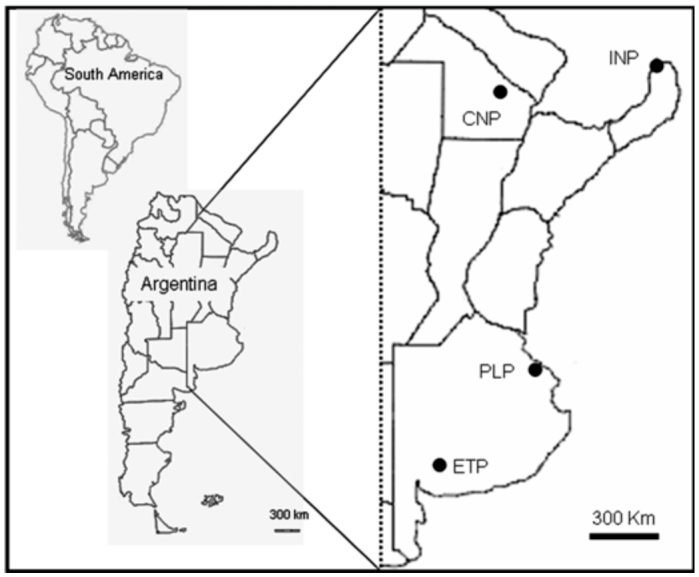
Location of sampling sites in Argentina, South America. Subtropical area, INP: Iguazú National Park (Misiones Province); CNP: Chaco National Park (Chaco Province); Temperate area, PLP: Punta Lara Provincial Park; ETP: “Ernesto Tornquist” Provincial Park (Buenos Aires Province). High quality figures are available online.

Field study was conducted from 1994 through 2009 in subtropical and temperate Argentina. The subtropical areas sampled were Iguazú National Park (25° 39′ S, 54° 18′ W) and Chaco National Park (26° 45′ S and 59° 37′ W), and the temperate areas were Punta Lara Provincial Park (34° 51′ S and 57° 52′ W), and Ernesto Tornquist Provincial Park (38° 10′ S and 62° 8′ W) (Figure 1).

Iguazú National Park is located north of Misiones Province, separated from neighboring Brazil by the Iguazú River. This park is part of the Paranense forest ecoregion (Dinerstein et al. 1995) with a topography and drainage pattern dominated by a basaltic plateau that reaches altitudes of 700 m. The annual rainfall varies from 1500 to 2000 mm, the dry season occurs in winter and abundant precipitations are recorded in summer. The mean temperature varies between 16 and 22° C. The dominant vegetation is the subtropical forest (APN 2009).

Chaco National Park is situated in Chaco Province and is part of the humid “Chaco” ecoregion (Dinerstein et al. 1995), a very gentle slope area dominated by depressed environments. It has a warm subtropical climate with summer rainfalls from 750 to 1300 mm. Within the protected area, forest environments, with savannahs, marshes and lagoons are found. The forest consists of trees that are 15 m tall, with the bottom layer covered by bromeliads with strong spines on the edge of their leaves, making the forest area almost impenetrable. Very dense areas of these formations (APN 2009) are seen on the rivers.

Punta Lara Provincial Park is situated on the Rio de La Plata, northeast of Buenos Aires Province. The park area corresponds to the austral part of the Paranense forest ecoregion (Dinerstein et al. 1995), which in this area is characterized by a narrow gallery forest on the river. Under natural circumstances, daily floods occur with the tide. On the other side of the gallery forest, there is an open field. Annual rainfall is approximately 850 mm, and the temperature is mild.

“Ernesto Tornquist” Provincial Park is situated southeast of Buenos Aires Province in the Austral Pampeana ecoregion (Dinerstein et al. 1995). This area is characterized by a plain with extensive pastures from which a mountain range of Paleozoic origin emerges. The mountains measure 170 km long with a maximum height of 1243 MASL and are oriented from northwest to southeast (Harrington 1947). The climate of the mountain is wet-subwet with scarce or null deficit of water (Burgos and Vidal 1951). Annual mean temperature and precipitations are 14° C and 896 mm (SMN 1981, 1986) with rains falling predominantly in the austral spring-summer period, and occasional snow falling in winter.

### Sampling

With the exception of bamboo, all phytotelmata were sampled by extracting the fluid contents with a pipette attached to a lift pump. After the first extraction, the plants were flushed twice with clean tap water, and the aquatic contents were extracted with a pipette after each wash. All insects were killed in the field and preserved in 80% commercial ethyl alcohol.

**Table 1.**
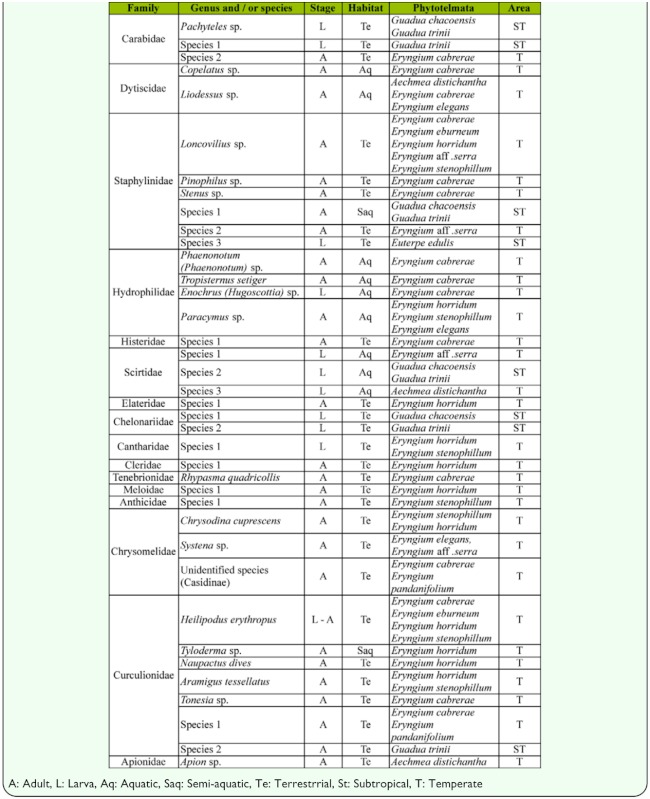
Coleopterns inhabiting phytotelmata (except trehole) at subtropical and temperate Argentina.

Due to the fact that the study was carried out in national parks where it was not possible to cut bamboo, samples were taken by making a hole with a drill in the middle of the internode and extracting the sample with a hose connected to a lift pump. Stump and broken bamboo were sampled again, and internodes were washed twice as with the other phytotelmata. Terrestrial beetles found on plants in the vicinity of phytotelmata were collected manually using entomological forceps.

## Results

The identified phytotelmata were: *Guadua trinii* (Nees) Ness ex Ruprecht, *Guadua chacoensis* (Rojas) Londoño and Peterson, *Merostachys clausseni* Munro (Poaceae), *Aechmea distichantha* Lemaire, *Aechmea recurvata* (Klotzsch), L.B. Smith, *Billbergia nutans* Wendland ex Regel, *Pseudananas sagenarius* (Arruda) Camargo, *Vriesea friburgensis* Mez, (Bromeliaceae), *Eryngium* sp. (Apiaceae), *Alocasia odora* (Lindl.) Hoch, *Philodendron bipinnatifidum* Schott ex Endlicher, (Araceae), *Urera baccifera* (L.) Gaudichaud-Beaupré ex Weddell, (Urticaceae), *Maranta* sp. (Marantaceae), *Euterpe edulis* Martius, (Arecaceae), *Dipsacus* sp. (Dipsacaceae) at Iguazú National Park, *Aechmea distichanta* and *Eryngium* sp. at Chaco National Park, *Eryngium horridum* Malme, *Eryngium stenophyllum* Urb., *Eryngium* aff. *serra* Cham. and Schltdl., *Eryngium elegans* Cham. and Schltdl. at Ernesto Tornquist Provincial Park, *Androtrichum giganteum* (Kunth) Pfeiff. (Cyperaceae), *Guadua trinii, Eryngium cabrerae* Pontiroli, *Eryngium eburneum* Decne, *Eryngium pandanifolium* Cham. and Schltdl., and *Dipsacus* sp. at Punta Lara Provincial Park. All except *Alocasia odora* and *Dipsacus* sp. are native phytotelmata.

**Figure 2.**
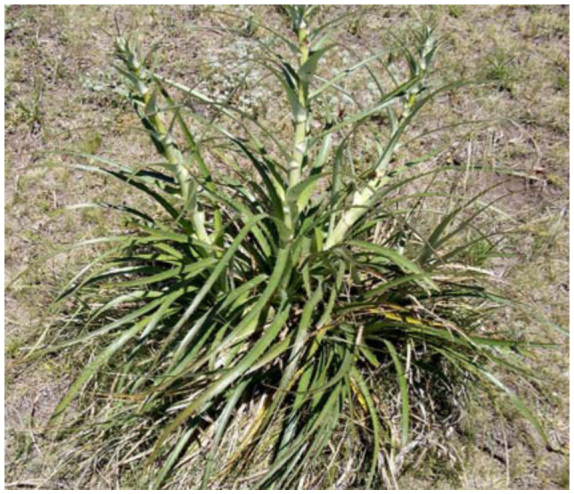
*Eryngium horridum* Malme (Apiaceae) at the field site in Sierra de la Ventana, Buenos Aires Province. High quality figures are available online.

**Figure 3.**
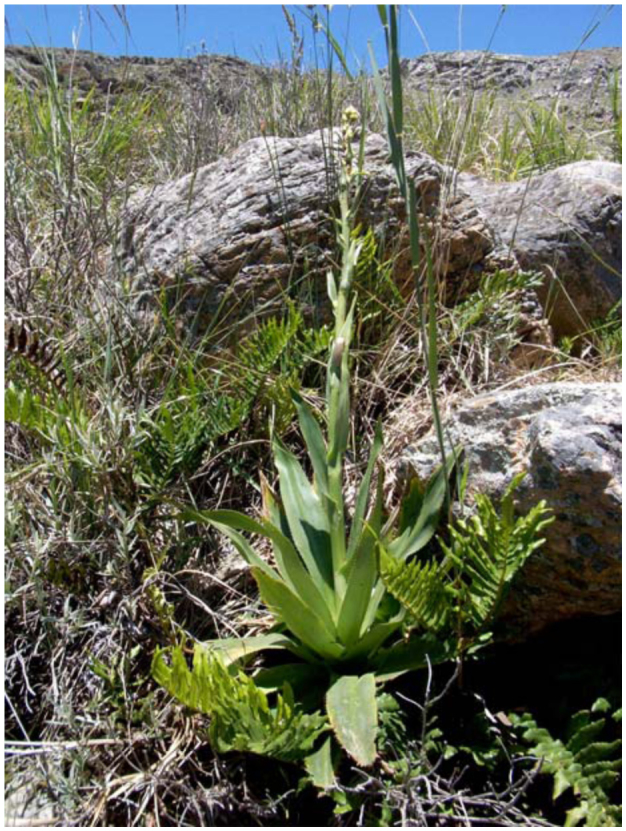
*Eryngium* aff. *serra* Cham. and Schltdl. (Apiaceae) at the field site in Sierra de la Ventana, Buenos Aires Province. High quality figures are available online.

From the above 21 classes of phytotelmata sampled, coleopterans were collected from *A. distichantha, E. edulis,* six species of *Eryngium* and two species of *Guadua* (48%) (Table 1). *Eryngium* species that are phytotelmata are morphologically similar to bromeliads (Figures 2, 3). The imbricate arrangement of their leaves delimits axils, as the peripheral leaves are older than the central ones. The internal axils hold free water and debris, the intermediate a semi-liquid interface composed mainly of debris and slime-fluxes, and the oldest, wet slime and debris. Thus, the real cavity where water is contained for a long time is limited to the axils of internal leaves. Water in external cavities is ephemeral and makes favorable habitats for semi-aquatic or terrestrial macro-invertebrates. Depending on the species, the size of the plants and the water retained by the axils was variable. The mean diameters were: *E. cabrerae* 83 cm (± SD 14.9); *E. horridum* 77 cm (± SD 0.17); *E. stenophyllum* 94 cm (± SD 0.23); *E.* aff. *serra* 46 cm (± SD 0.15); *E. elegans* 40 cm (± SD 0.11). The volumes of impound water were: 69.4 ml (± SD 43.1); 20.6 ml (± SD 14.2); 7.4 ml (± SD 6.6); 19.1 ml (± SD 17.1); 19.1 ml (± SD 17.1); 7.7 ml (± SD 12.3), respectively. No measures of *E. eburneum* were recorded.

**Figure 4.**
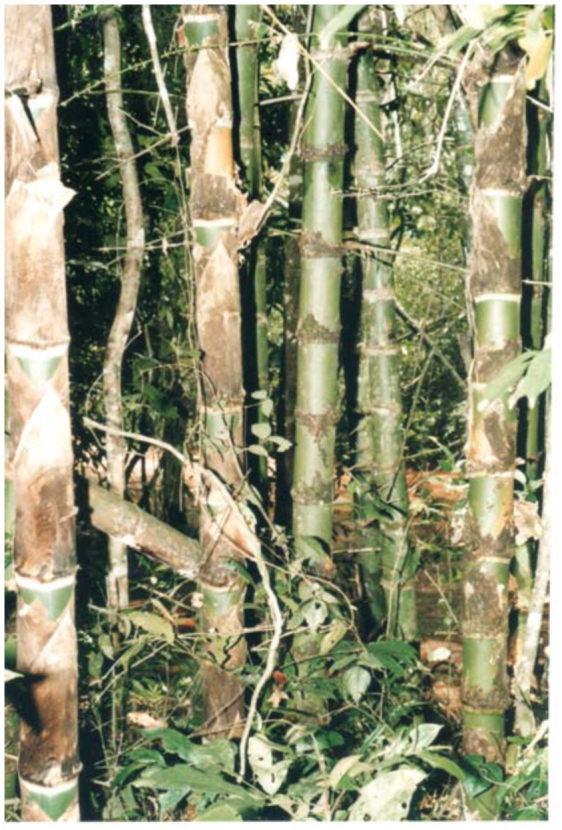
*Guadua chacoensis* (Rojas) Londoño and Peterson (Poaceae) at the forest site in Iguazú National Park, Misiones Province. High quality figures are available online.

**Figure 5.**
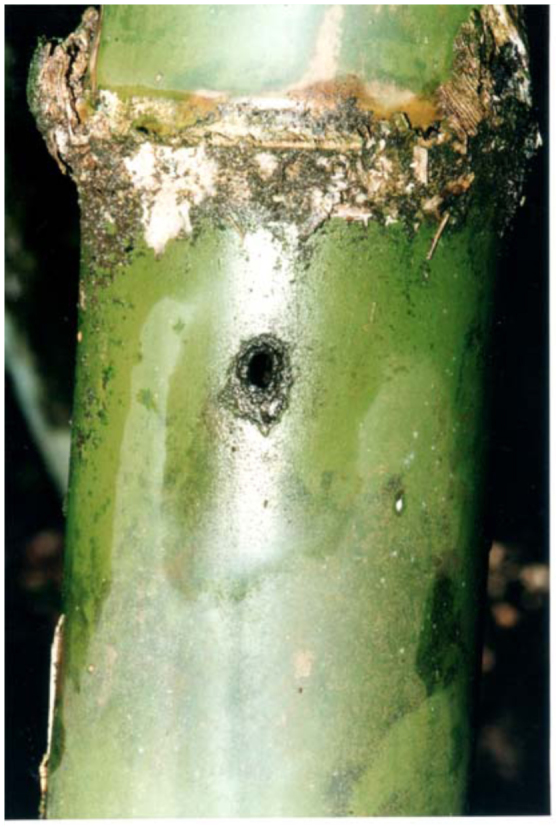
Internodes of *Guadua chacoensis* (Rojas) Londoño and Peterson (Poaceae) with a hole in the wall. High quality figures are available online.

**Figure 6.**
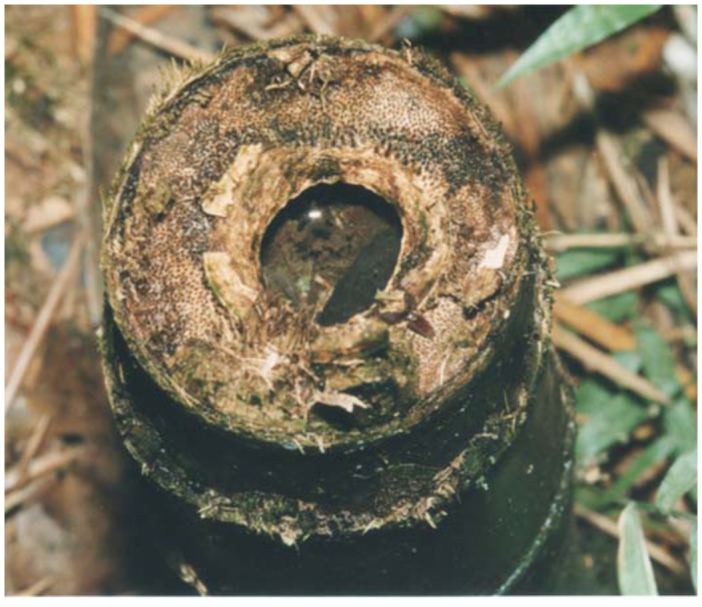
Stumps of *Guadua chacoensis* (Rojas) Londoño and Peterson (Poaceae). High quality figures are available online.

*Guadua* bamboos (Figure 4) grow mainly in low places near streams, and can reach up to 12 meters. In the study area we observed two types of bamboo phytotelmata, one in the internodes that have one or more holes in the wall by the penetration of rain water (Figure 5) and the other in the stumps as a result of cutting bamboo (Figure 6). The longitude of the internodes, inlet holes and the volume of water retained into the internodes were different in each species of bamboo. *Guadua chacoensis* was the biggest, with a mean longitude, hole diameter and water volume of 23.3 cm (± SD 3.5), 9.1 mm (± SD 4.9) and 366.1 ml (± SD 225), while *G. trinii* was 23.1 cm (± SD 5.5), 6.1 mm (± SD 3.8) and 45.2 ml (± SD 41.1) respectively.

**Figure 7.**
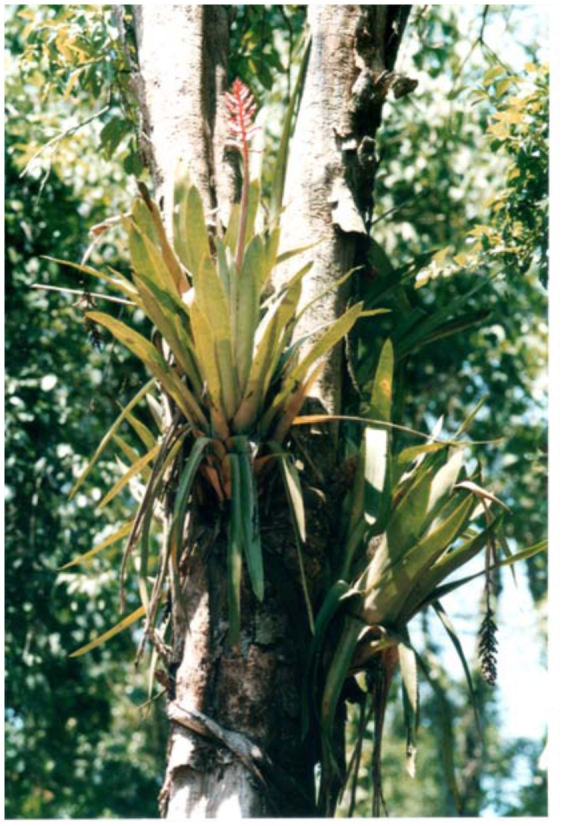
*Aechmea distichantha* Lemaire (Bromeliaceae) at the field site in Iguazú National Park, Misiones Province. High quality figures are available online.

*Aechmea distichantha* (Figure 7) is epiphytic or terricolous and was the dominant bromeliad in Chaco and Iguazú subtropical parks. The volume of water impounded in its axils, sometimes several liters, was much greater than the volume in the axils of *Eryngium.*

**Figure 8.**
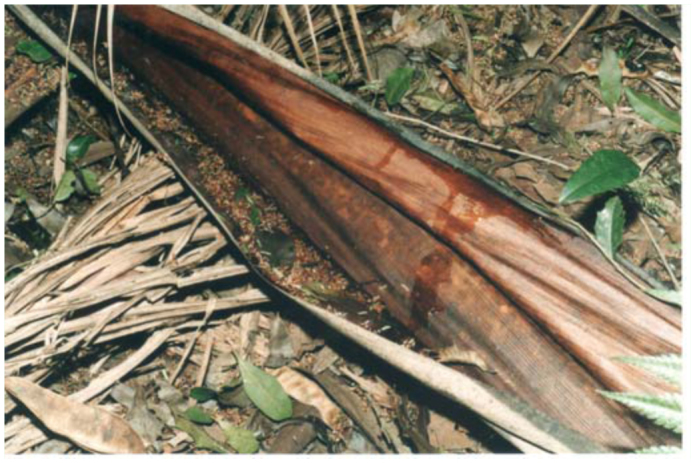
Fallen floral bracts of the palmetto *Euterpe edulis* Martius (Arecaceae) at the field site in Iguazú National Park, Misiones Province. High quality figures are available online.

The fallen floral bracts of the palmetto *Euterpe edulis* (Figure 8) were the only type of phytotelmata found in this study in non-living parts of the plant. The bracts, which collect rain water in their inner cavity, are woody and more than one meter in length.

Sixteen families of coleopterans were recorded, fourteen from *Eryngium*, five from *Guadua*, three from *A. distichantha*, and one from *E. edulis.* Hydrophilidae, Histeridae, Elateridae, Cantharidae, Cleridae, Tenebrionidae, Meloidae, Anthicidae and Chrysomelidae were present exclusively in species of *Eryngium*, while chelonariids were found in *Guadua*, and Apionidae in *A. distichantha.* No exclusive family was found in *E. edulis.* Dytiscids were present in *Eryngium* and *A. distichantha*; scirtids in *Eryngium*, *Guadua* and *A. distichantha*; staphylinids in *Eryngium*, *Guadua* and *E. edulis*, and curculionids in *Eryngium* and *Guadua.* Among aquatic coleopterans, *Enochrus (Hugoscottia)* sp. (Hydrophilidae) and unidentified Scirtidae were collected in the larval stage, while *Copelatus* sp., *Liodessus* sp. (Dytiscidae), *Phaenonotum* sp., *Paracymus* sp., *Tropisternus setiger* (Hydrophilidae) were collected as adults (Table 1). Semi-aquatic species were represented by Chelonariidae larvae and Curculionidae by *Tyloderma* adults (Table 1).

Families of terrestrial beetles were Carabidae, Staphylinidae, Histeridae, Tenebrionidae, Elateridae, Cantharidae, Cleridae, Meloidae, Anthicidae, Chrysomelidae and Curculionidae (Table 1). The weevil *Heilipodus erythropus* (Klug) (Coleoptera, Curculionidae) was the only species collected in both larval and adult stages from *Eryngium horridum.* Larvae of this weevil were observed drilling the stalk of inflorescence and adults living in axils.

**Figure 9.**
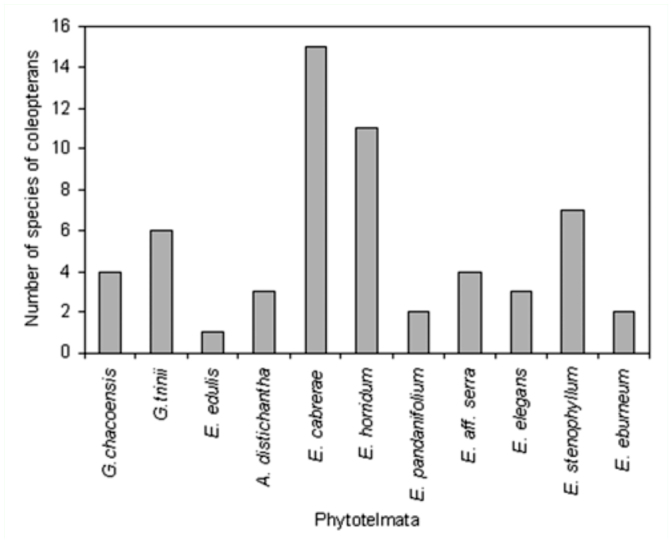
Number of species of coleopterans by class of phytotelmata from subtropical and temperate Argentina. High quality figures are available online.

The greatest diversity of coleopterans (27 species) was found in *Eryngium* (Table 1); the largest percentage of species was collected from *E. cabrerae* (56%), followed by *E. horridum* (41%), *E. stenophyllum* (26%), *E.* aff. *serra* (15%), *E. elegans* (11%), and *E. eburneum* and *E. pandanifolium* (7%). Beetles were poorly represented in *E. edulis*, *A. distichantha* and in both *Guadua* bamboos (Figure 9). No species inhabiting *Eryngium* were observed in *Guadua* or *E. edulis.* Only *Liodessus* sp. was found both in *Eryngium* and in *A. distichantha* (Table 1).

## Discussion

Among the 21 classes of phytotelmata reported here, 15 correspond to the subtropical northern area, and nine to the temperate area. Coleopterans were only recorded in *Eryngium*, *Guadua*, *A. distichantha* and *E. edulis.* The species of *Eryngium* are dominant in temperate areas (Campos 2010), while the species of *Guadua* are distributed in the subtropical area, extending in the temperate area along a narrow strip of forest on La Plata River. *Aechmea distichantha* and *E. edulis* are subtropical.

Five families of aquatic coleopterans have been related to phytotelmata in the Neotropical region. A recent review carried out by Frank and Lounibos (2008) cites representatives of the families Scirtidae, Dytiscidae and Hydrophilidae inhabiting bromeliads. Other phytotelmata, like bamboo and fallen flower bracts of a palm, also include a few species of the families Elmidae (Sanchez and Liria 2009) and Noteridae (Greeney 2004), respectively. On the other hand, the diversity of terrestrial beetles that visit phytotelmata is higher. A study carried out in southern Brazil (Mestre et al. 2001) shows that the bromeliad *Vriesea inflata* (Wawra) Wawra is visited by species of 13 families of terrestrial coleopterans. However, other authors (Gutierrez Ochoa et al. 1993; Greeney 2001; Frank et al. 2004) mentioned other families not cited by Mestre et al. (2001) that use bromeliads and other phytotelmata to hide or find prey, summarizing at least 20 families. In accordance with Frank and Lounibos (2008), representative species of three families of aquatic coleopterans were recorded from phytotelmata of Argentina other than bromeliads. In contrast with the numerous terrestrial coleopterans known, only species of 12 families were recorded, probably due to the lack of an exhaustive search. Several studies show that coleopterans are a taxon of scarce richness and abundance in the communities living in phytotelmata. The exceptions were observed in invertebrate communities of the bromeliads *Vriesea extensa* Sm. (Synon.: *Alcantarea extensa* (Sm.) Grant) and *Vriesea inflata* where a Carabidae (Juncá and Borges 2002) and a Scirtidae (Mestre et al. 2001), respectively, were dominant although richness was low, contrary to the high species richness (in particular Dytiscidae) observed in fallen floral bracts of a palm (Greeney 2004). In our study, although not quantified, the small number of beetles was highly contrasting with dipterans and other arthropods in all sampled phytotelmata.

### Aquatic Coleopterans

Dytiscidae — Species of four genera of Dytiscidae have been cited in the literature as inhabitants of phytotelmata (*sensu lato*): *Copelatus* Erichson and *Desmopachria* Babington in bromeliads (Greeney 2001; Frank and Lounibos 2008) and in fallen flower bracts of the stilt-root palm *Iriartea deltoidea* Ruiz and Pavon (Arecaceae: Iriarteeae), and *Laccophillus* Leach and *Thermonectus* Dejean in this last phytotelmata (Greeney 2004). In our study, adults of unidentified species of the genus *Copelatus* and *Liodessus* Guignot dwelling in the axils of *Eryngium* and *Liodessus* and in the axils of *A. distichantha* were found. As adults were scarce and no immatures were found, it is presumed that these could be opportunistic species, which use water from the axils when the pools dry up.

Hydrophilidae — There are two subfamilies with species that inhabit phytotelmata, Sphaeridiinae and Hydrophilinae. Subfamily Sphaeridiinae is found in a great diversity of habitats, mainly terrestrial, but in some cases some species of this subfamily are secondarily aquatic, while Hydrophilinae are mostly aquatic and are found in most freshwater environments (Archangelsky 2004). All the species specialized in living in Bromeliaceae are Sphaeridiinae subfamily and belong to the genera *Coelostoma* Brullé, *Lachnodacnum* Orchymont, *Phaenonotum* Sharp, *Omicrus* Sharp (Frank and Lounibos 2008). Other members of this subfamily are *Dactylosternum* Wollaston and *Pelosoma* Mulsant, which were found inhabiting in fallen flower bracts of a palm (Greeney 2004). The genera of Hydrophilinae that were reported from phytotelmata are *Hydrobiomorpha* Blackburn, *Enochrus* Thomson and *Derallus* Sharp, all collected as adults from fallen floral bracts of a palm (Greeney 2004). The only record of a hydrophilid inhabiting treeholes in the Neotropic region was quoted by H. F. Greeney as unpublished data in “The insects of plant-held water: a review and bibliography” (Greeney 2001), and did not mention the genus. During our sampling, we found *Tropisternus setiger* (Germar), *Paracymus* sp. (adults) and *Enochrus* sp. (larva), all Hydrophilinae, and *Phaenonotum* sp. (adults) a Sphaeridiinae, living in the axils of four *Eryngium* phytotelmata. *T. setiger* is a ubiquitous species that lives in ephemeral (Campos et al. 2004), temporary, and permanent pools (Von Ellenrieder and Fernández 2000) and therefore it cannot be regarded as a specific inhabitant of phytotelmata. Its habit of oviposition on aquatic vegetation (Jerez and Moroni 2006) limits its possibility of developing in the axils of *Eryngium* or other phytotelmata. *Enochrus* is a widely distributed genus living in temporary and permanent environments, whose females lay egg cases that adhere to the underside of the leaves of the floating *Azolla filiculoides* Lamarck (Salviniales: Azollaceae) (Fernández 1992). *Eryngium* phytotelmata are frequently partially submerged in floodwater puddles, where dense vegetation is formed dominated by *A. filiculoides.* When the pond dries up this small plant is retained in the axils (Campos unpublished data), and can contain larvae or egg cases of *Enochrus* between its leaves. This would explain the presence of *Enochrus* larvae in the leaf axils of *E. cabrerae* as was observed in this study.

Scirtidae — Is the most abundant family of coleopterans that inhabit phytotelmata, mainly treeholes (Greeney 2001), bamboo (Louton et al. 1996), and bromeliads (Ospina-Bautista et al. 2004). There are five genera known from phytotelmata: *Scirtes* Illiger (Picado 1913), *Cyphon* Paykull, *Ora* Clark (Frank et al 2004), *Prionocyphon* Redtenbacher (White 1978), and *Flavohelodes* Klausnitzer (Stribling and Young 1990), whose larval habits are mainly detritivorous converting the leaf litter into fine particles which benefit fine particle feeders co-occurring in the same phytotelmata (Daugherty and Juliano 2003). Scirtid species from South America are little known, and the only genera cited as phytotelmata inhabitants are *Scirtes*, and *Cyphon* collected from Bromeliaceae in Colombia (Ospina-Bautista et al. 2004) and Venezuela (Liria 2007), respectively. Other studies carried out in Brazil (Mestre et al. 2001), Perú (Louton et al. 1996) and Argentina (Montero et al. 2010) mentioned Scirtidae living in bromeliads and bamboo without indicating their identity. In this study, larvae of three morpho-species living in *Guadua, A. distichantha* and *Eryngium* phytotelmata in both subtropical and temperate areas were found. However, they could not be identified because they were not reared to the adult stage (and because there are no keys to Argentinian scirtid larvae).

### Hydrophilic or semi-aquatic Coleopterans

Chelonaridae — This family is little known and has been found in association with treeholes (Spangler 1980) and bromeliads, apparently feeding on decaying plant matter (Costa et al. 1988). However, there is still controversy over whether or not their larvae are aquatic. Spangler (1980), in contrast with others authors (Boving and Craighead 1931), concludes that these larvae are not aquatic because they do not have real anal gills and attributes their presence in aquatic environments as a consequence of the moss being washed off of their habitat. Only a few larvae are known and little is known of their behavior. More research could reveal unknown species that inhabit phytotelmata that could be adapted to aquatic life. In this study, we reported unidentified Chelonaridae larvae inhabiting bamboo internodes (*Guadua chacoensis* and *G. trinii*). The presence of these larvae in a habitat of vertical and smooth walls, such as bamboo internodes where there is not an interface between water and decaying matter (as in axils of bromeliads and treeholes), would suggest that these species are better adapted to live in an aquatic environment. Because the behavior of Chelonaridae species is unknown, this hypothesis should be tested.

Curculionidae - Of the 131 species of semi-aquatic weevils (Curculionidae, Erirhinidae and Dryophthoridae) known from Argentina (Morrone and O'Brien 1999), we collected an unidentified specie of the genus *Tyloderma.* Lanteri et al. (2002) reports that 12 species of *Tyloderma* in Argentina are associated with aquatic macrophytes. We found abundant individuals of *Tyloderma* living in axils of *E. horridum*, but none was immersed in water.

### Terrestrial Coleopterans

This group is composed of beetles that use phytotelmata as refuge or searching for prey, and as already mentioned, is represented by many families. Some species are closely associated with the plant host, while others are occasional. The families that were found during our study are discussed below.

**Figure 10.**
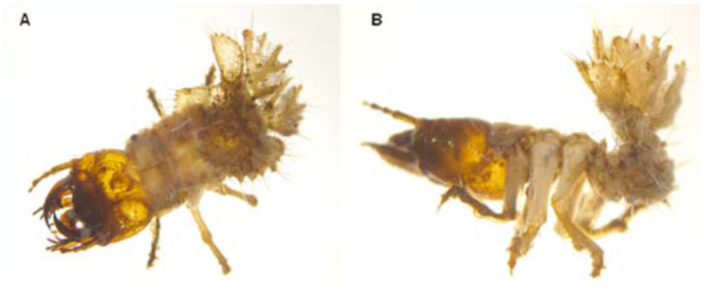
Habitus of larva of *Pachyteles* sp. (Carabidae), A: dorsal ; B: lateral. High quality figures are available online.

Carabidae — The species which live associated with phytotelmata were only mentioned as inhabitants of Bromeliaceae and belong to *Agra* Fabricius, *Calathus* Bonelli, *Callida* Latreille and Dejean, *Colpodes* MacLeay, *Lebia* Latreille, *Lia* Eschscholtz, *Onypterigia* Dejean, *Phloeoxena* Chaudoir, *Pterostichus* Bonelli, (Laessle 1961; Zaragoza 1974; Gutiérrez et al. 1993) and *Platynus* Bonelli (Montes de Oca et al. 2007) that were collected in Jamaica and Mexico. Less specialized Carabidae were found occasionally, looking for prey in fallen floral bracts of a palm in Ecuador (Greeney 2004). During this study, a conspicuous larva of the genus *Pachyteles* Perty, characterized by a widened eighth abdominal segment and sensory bristles that work like a trap when capturing prey, was found in the internodes of *Guadua* bamboo. This eighth modified segment (Figure 10) is used to plug the opening of the gallery in which they live (Costa et al. 1988). Our observations revealed that the larvae of *Pachyteles* sp., reared in bamboo internodes that contained the original sediment and water, dived to the bottom, from which they extracted debris with their mandibles and built a cell compartment on the inner wall of the internodes. It was verified that they are good predators of Isoptera and other small insects because, when feeding, the body bends towards the last abdominal segment and adopts a V form (Costa et al. 1988). It was also observed that the community of macro-invertebrates living in bamboo internodes is very diverse (unpublished data), and the free water space is occupied by various terrestrial insects, even by termite and ant nests. For this reason, *Pachyteles* larvae could be considered one of the top predators within this community. On the other hand, the unique individual of the unidentified Carabidae collected from *Eryngium cabrerae* is a terrestrial species considered a visitor.

Staphylinidae — This familiy is represented in communities of bromeliads (e. g. Picado 1913; Lüderwaldt 1915; Zaragoza 1974), fallen floral bracts of a palm (Greeney 2004), and from inflorescences of several species of *Heliconia* L. (Zingiberales: Heliconiaceae) (Frank and Barrera 2010). Six species of Staphylinidae were collected (Table 1). *Loncovilius* sp., *Pinophilus* sp. and *Stenus* sp. were present exclusively in the axils of the species of *Eryngium. Loncovilius* sp. was most abundant mainly in *Eryngium cabrerae.* An unidentified adult and larva were collected from species of *Guadua* and from fallen floral bracts of *E. edulis*, respectively. All species could be considered as potential top predators from the aquatic communities because they can prey on various species of aquatic larvae as was observed by Greeney (2004) and Frank and Barrera (2010) during studies carried out in communities inhabiting the fallen floral bracts of a palm, and flower bracts of *Heliconia*, respectively.

Histeridae - The only species of this family associated with phytotelmata, *Omalodes sobrinus* Erichson, was reported from *Bromelia hemisphaerica* Lamarck 1783 of Mexico, and was listed as a very scarce herbivore (Gutiérrez et al. 1993). We reported an unidentified species associated with *E. cabrerae* in temperate areas, which was also of very low abundance.

Elateridae - Few studies have been done on Elateridae associated with phytotelmata. Zaragoza (1974) cited Elateridae inhabiting phytotelmata to species level. The species cited were: *Platycrepidius boucardi* (Salle), *Megapenthes cincticollis* Champion, *Ischiodontus anceps* Candèze, and *Anchastus seminiger* Champion, inhabiting the bromeliad *Aechmea bracteata* (Swartz) Grisebach in Mexico. Domingues et al. (1989) reported larvae of unidentified Elateridae inhabiting epiphytic bromeliads in Brazil and Mestre et al. (2001) found unidentified adults of Elateridae in the epiphytic *Vriesea inflata* (Bromeliaceae), but not in terrestrial ones. While Frank et al. (2004) reported *Conoderus amplicollis* (Gyllenhal) inhabiting *Tillandsia recurvata* (Linnaeus) L. in USA. We reported an unidentified species associated with the terrestrial *E. horridum* (Apiaceae).

Cantharidae - The only record of Cantharidae is *Chauliognathus tripartitus* Chevrolat inhabiting *Vriesea* sp. from Mexico (Zaragoza 1974). In our study, we found few larvae of unidentified Cantharidae inhabiting two species of *Eryngium* that could be considered occasional predators that use phytotelmata to hide in or find prey.

Cleridae and Meloidae — This is the first record of representatives of these families of coleopterans associated with phytotelmata. We found adults in *E. horridum*, but unfortunately, their identification was not possible.

Tenebrionidae - There are few reports of associated Tenebrionidae to phytotelmata, all of them referring to Bromeliaceae. *Goniadera oculata* Champion, *Eurymepoton* sp. and *Eleodes* sp. were cited inhabiting *A.* *bracteata*, *Tillandsia caputmedusae* Morren, and *Vriesea chiapensis* Matuda, respectively (Zaragoza 1974). *Stibia* sp. was found associated with *Bromelia hemisphaerica* (Gutiérrez et al. 1993) and *Glyptotus cribratus* LeConte to *Tillandsia fasciculata* Swartz, the latter in the Nearctic region (Frank et al. 2004).

Another unidentified tenebrionid was found in terrestrial and epiphytic *Vriesea inflata* (Mestre et al. 2001). The ecological niche of the phytotelmic species is unknown; however, Zaragoza (1974) classified them as detritivorous because they feed on the debris that accumulates in the leaf axils. In the present study, we report *Rhypasma quadricollis* (Fairmaire) associated with *Eryngium cabrerae*, a phytotelmata different from bromeliads.

Anthicidae - The only species of this family reported from phytotelmata is *Anthicus asphaltinus* Champion, collected in Mexico from *B. hemisphaerica*, which was considered scarcely abundant and its ecological niche unknown (Gutiérrez et al. 1993). We collected an identified specimen from *E. stenophyllum.*

Apionidae: The genus *Apion*, classified as Curculionidae and Brentidae by other authors, was reported from *A. bracteata*, *Vriesea chiapensis* (Bromeliaceae) (Zaragoza 1974), and *T. fasciculata* (Frank et al. 2004). We report a species of the same genus inhabiting *A. distichantha*, in the subtropical area.

Chrysomelidae — Although most of the Chrysomelidae are terrestrial, some (Donaciinae, Chrysomelinae and Galerucinae) exhibit some degree of adaptation to aquatic life (Konstantinov 2003). The few species of Chrysomelidae reported as inhabitants of phytotelmata are of terrestrial habits and feed on the leaves of the host plant (Frank and Lounibos 2008). *Acentroptera pulchella* Guérin-Méneville (Mantovani et al. 2005) of Brazil, *Calliaspis rubra* (Olivier) of Peru (Burgess et al. 2003), and *Pachybrachys* sp. of Mexico (Gutierrez et al. 1993) were reported from Bromeliaceae, while some species of *Systena* Chevrolat were found associated with *Eryngium* (Apiaceae) (Jolivet and Hawkeswood 1995). No aquatic Chrysomelidae were reported from any phytotelmata. The three species collected during the present study were associated with *Eryngium* spp. One of these was an unidentified cassidine; the others, *Chrysodina cuprescens* Boheman and *Systena* sp., are polyphagous, but only the latter was cited feeding on the leaves of *Eryngium* (Jolivet and Hawkeswood 1995).

Curculionidae — This family is the most extensive of the terrestrial coleopterans associated with phytotelmata, and its species were reported mainly from Bromeliaceae, a major pest of these plants (Frank 1999). In their 2002 review, Lanteri et al. reported that *H. erythropus* and *Tonesia argentinica* Hustache were the only curculionid associated with *Eryngium* species in Argentina. We also report, *Napactus dives* Klug associated with *E. horridum*, and *Aramigus tessellates* (Say) associated with *E. horridum*, *E. stenophyllum* and *E. cabrerae.* We also report an unidentified species of Curculionidae from *Guadua* bamboo. *Heilipodus erythropus* is a species extensively distributed in Argentina and neighboring countries (Lanteri et al. 2002). It was the most abundant in our study and was collected from five species of *Eryngium.* Its larvae drill the inside of the stem of the inflorescence of *Eryngium paniculatum* Cavanilles and Dombey, where they develop their life cycle and later pupate in the root (Ríos de Saluso 1997). De Saluso mentioned that this weevil was found exclusively on *Eryngium*, but an old report cited this Curculionidae causing damage to Bromeliacaea (Silva et al. 1968). Although *H. erythropus* is not cited by Morrone and O'Brien (1999) as a semi-aquatic species, it was observed in the field that when the adult is disturbed, it dives into the water where it remains for several minutes until the danger disappears (Campos unpublished data). This behavior was observed several times, even without touching the plant. The mere shadow of our approach made them take refuge in the water. *Tonesia argentinica* was reported inhabiting *E. paniculatum* in subtropical Chaco Province. We found an identified species of this genus associated with *E. cabrerae* in temperate Buenos Aires Province.

Species of Elateridae, Meloidea, Chrysomelidae, and Curculionidae were observed not only in the axils of *Eryngium* phytotelmata, but also on inflorescence during the hottest hours, taking refuge in the axils in the morning or during drier periods. These species might not be interacting in the aquatic community, except for their input of organic matter as a consequence of their stools.

The terrestrial beetle community associated with phytotelmata in the subtropical and temperate region of Argentina was richer in species than the aquatic community. Most species of terrestrial beetles found in this study are ubiquitous, being associated with other plants that are not phytotelmata. However, some staphylinids species seem to be exclusive inquilines of *Eryngium*, interacting with the aquatic community as predators. Another specific beetle is the weevil *H. erythropus* whose life cycle takes place in different parts of the plant. Among the aquatic beetles that inhabit *Eryngium*, none of them was specific. All the species found in this study use aquatic environments other than phytotelmata. A possible hypothesis is that the presence of ubiquitous species in phytotelmata could be a strategy of displacement to other aquatic environments, using these plants occasionally and for a short time. Richness of beetles in *Guadua* bamboo was low, however the most common and abundant inhabitant was the carabid *Pachyteles.* For their behavior and their habit of constructing cells within internodes, one could hypothesize that it is a species closely associated with this type of micro-habitat interacting with phytotelmata through the removal of sediment and providing nutrients as a product of excrements. On the other hand, the scarcity of water beetles could be due to the relative smallness of the inlets which, being located in the side wall of the internodes, could not be detected by most ubiquitous species. The only water beetles found in the stumps were scirtids, which are also common inhabitants of treeholes. Their presence could be due to the similarity of bamboo stumps with treeholes.

The number of coleopterans associated with phytotelmata in Argentina may increase with the exploration of the subtropical forest of the Yungas, located in the northwest. We believe that the same will happen with the diversity of plants that function as phytotelmata. However, the development of comprehensive lists could be limited by the scarce taxonomic knowledge of the Neotropical coleopterans.
